# Conceptualizing the peer contribution in Open Dialogue practice

**DOI:** 10.3389/fpsyg.2023.1176839

**Published:** 2023-08-17

**Authors:** Corrine Hendy, Jerry Tew, Sarah Carr

**Affiliations:** ^1^Nottinghamshire Healthcare Foundation Trust, Nottingham, United Kingdom; ^2^Mental Health and Social Work, School of Social Policy, University of Birmingham, Birmingham, United Kingdom; ^3^Department of HSPR, Institute of Psychiatry, Psychology and Neuroscience, King’s College London, London, United Kingdom

**Keywords:** Open Dialogue, peer support, practice principles, attunement, validation, connection and mutuality, self-disclosure

## Abstract

In English mental health services, people with their own experience of mental distress have trained as Open Dialogue practitioners and have been employed as peer practitioners, co-working as equals alongside workers with professional backgrounds in Network Meetings. The conceptual underpinnings of the peer practitioner role have been drawn from the principles and relational approach of Intentional Peer Support. These have significant similarities with Open Dialogue, in terms of philosophical and theoretical orientations, with a particular focus on what happens in the “between” of a relational encounter. However, there are also significant differences in how practice principles are conceptualized, particularly around areas such as mutuality and self-disclosure. This article offers an analysis of this conceptual territory drawing on the relevant literature. This is then taken forward with the teasing out of specific practice principles that capture the unique contribution that peer practitioners can bring to Open Dialogue practice. These are derived through discussions that took place in an Action Learning Set for peer practitioners who have been involved in delivering Open Dialogue services in mainstream mental health service settings. This was part of a wider research study entitled *Open Dialogue: Development and Evaluation of a Social Network Intervention for Severe Mental Illness (ODDESSI).* The principles address how peer practitioners may be particularly well-placed to offer attunement, validation, connection and mutuality, and self-disclosure – and hence how they may be able to contribute an additional dimension to dialogical practice.

## Introduction

In England, the development of Open Dialogue has received strong support from many who have used, or are using, mental health services, as well as from family, friends and practitioners. It is seen as offering a more open and inclusive way of working with mental distress. In many instances, Open Dialogue is being introduced into services where the role of peer workers is also being developed – opening up new opportunities, but also raising certain challenges in terms of how the two approaches might best be integrated. In the United States, peer workers were integral team members in the roll-out of the Open Dialogue inspired Parachute NYC in New York. Despite some challenges with structural constraints around the introduction of peer specialists, this project established the principle that they should be considered as equal practitioners, rather than as support workers assigned to practical tasks outside of Network Meetings (Hopper et al., 2020; Wusinich et al., 2020). Internationally, we have seen other developments of Peer supported Open Dialogue practice (see for example, Lorenz-Artz et al., 2023).

Within the United Kingdom, the Peer Practitioner role has been a key component within some Open Dialogue teams since 2014, and peers have trained and become accredited Open Dialogue practitioners or therapists, with the potential to effect positive clinical outcomes and experiences (Razzaque and Stockmann, 2016; Kinane et al., 2022). The first national multi-site trial evaluating Open Dialogue in English NHS mental health services – Open Dialogue: Development and Evaluation of a Social Network Intervention for Severe Mental Illness (ODDESSI) – has included an explicit commitment to peer practitioner involvement in multidisciplinary teams (Pilling et al., 2022). Peer supported Open Dialogue (POD) may be seen as a variation of the Open Dialogue approach as originally developed in Western Lapland and guided by the same principles, with the added contribution of peer practitioners who have their own experiences of crisis, mental distress and personal recovery, and (in many instances) of using mental health services.

The development of peer worker roles in mental health services has often been somewhat *ad hoc*, with insufficient thinking about the nature of the role and how this should be supported. In particular, there can be some confusion and inconsistency between peer support and peer practitioner roles in services and teams (Grey, 2019). This has resulted in experiences and outcomes – both for service users and for the workers themselves – which have not always been entirely positive (Gillard and Holley, 2014; Vandewalle et al., 2016). Both internationally and in the United Kingdom, the more focused peer practitioner roles have often been inspired by the Intentional Peer Support (IPS) model (Mead and MacNeil, 2006; Grey, 2019). IPS offers a way of building purposeful relationships between people who have direct experience of mental distress. It is a process where both parties use the relationship to look at the meaning of their experiences from new angles, develop greater awareness of personal and relational patterns, and to support and challenge each other. It is a practice that is fundamentally dialogical – and hence has the potential to provide a good “fit” with the principles of Open Dialogue. For example, as Kemp et al. (2020) observe, “the OD principle of ‘tolerating uncertainty’ is not entirely different to peer support principles of ‘not knowing’ and ‘dignity of risk’, which support self-determination and seek to avoid risk-averse practice” (p. 58).

While the Treatment Principles that define Open Dialogue practice are well established (Seikkula et al., 2003), there is now an opportunity to revisit these, in conjunction with those of IPS, in order to clarify the conceptual underpinnings of the emergent peer practitioner role in Open Dialogue. In this Paper, we start to map out what this might look like, drawing both upon the relevant philosophical and practice related literature, and on discussions that took place in an Action Learning Set for peer practitioners who have been involved in delivering Open Dialogue services linked to the ODDESSI research trial (Pilling et al., 2022). We hope that this can better articulate “a coherent and profound narrative” about POD, and what this may contribute to the development of dialogic practice (Lorenz-Artz et al., 2023).

## Conceptual starting points

Alongside Tom Anderson’s reflecting teams family therapy approach (Andersen, 1995), Open Dialogue represents a decisive break with earlier systemic practice in which “expert” conversations took place behind a one-way screen in parallel with the therapeutic conversation taking place with (and between) family members (Sidis et al., 2022). The function of these conversations was to generate hypotheses and formulations, using the combined inspirations of a supervisory team to outmaneuver resistances to change within the organization of a family system. Crucial to the break was a shift from a hierarchically organized discourse to a democratic practice of an “open” dialogue in which all conversation takes place in the room and draws upon the language and forms of expression that are being used by the person experiencing mental distress and those who were part of their relational network. As Jakko Seikkula writes,

“Perhaps as therapists we are so used to thinking so much about being skillful in methods and interventions that it is difficult to see the simplicity. All that is needed is to be present and to guarantee that each voice becomes heard” (2008, p. 489).

This conception of dialogue harks back to the existentialist idea of the authentic “I Thou” encounter as originally described by Buber (2000), with its focus on the potential for something new to emerge in the “between” of an encounter that is more than the individual contributions of those involved, and which has the potential to shift the experience (and understanding) of self *and* other.

There are similar echoes of the “I Thou” encounter in how dialogue is conceptualized in Intentional Peer Support. As Shery Mead writes,

“In real dialogue, we are able to step back from our truth and be very deeply open to the truth of the other person while also holding onto our own. When this type of dialogue occurs, both of us have the potential to see, hear, and know things in ways that neither of us could have come to alone” (Mead, 2014, p. 8).

She explicitly focuses on the importance, and creativity, of the “between” space:

“When we pay attention to the relationship … we are paying attention to what is going on between us. In other words, we focus on the “space” between us, what is happening right here, right now that can either move us forward or back…. When I pay attention to what’s going on between us, it opens up a line of communication that supports honesty, safety, integrity, and ultimately changes the very direction I had wanted to go without you” (Mead, 2010a, p. 13).

A pre-requisite for dialogue is taking time to establish an authentic personal connection. In IPS, this is described as “the bond that is created when people feel genuinely understood and trusting enough to go deeper” (Mead and Filson, 2017, p. 147). For Open Dialogue, cultivating such a connection with the person is equally crucial. International research on peer supported Open Dialogue shows that many peer practitioners believe the idea of “peer” to be about relationships rather than roles or identities (Grey, 2019). Mary Olson and colleagues argue that there should be no “ready-made solutions” or “pre-planned interventions” in Open Dialogue (Olson et al., 2014, p. 27). This strongly relates to the practice principle of tolerance of uncertainty (Seikkula et al., 2003), and an intention to keep the focus on “connection – as opposed to direction” (Razzaque and Stockmann, 2016, p. 352).

For Mead, there is a crucial distinction between peer support more generally, which may involve *helping* the other, and Intentional Peer Support which is about a fundamentally mutual process of *learning* with and from each other:

“Learning implies a curiosity, an inquisitiveness about the other, their way of knowing, their way of making sense of the world, whereas helping often implies that you already have the answers, that you know better, that you can come in and tell someone what to do” (Mead, 2010a, pp. 12–13).

This may not happen immediately, and time may be needed for people to become connected enough to allow the expression of emotionally charged experiences, after which there may be an emergence of a “between” space in which it may be possible to develop “a new ‘shared’ story” (Mead and Hilton, 2003, p. 89).

Although Open Dialogue and IPS may share some common conceptual roots, there can be some tensions as to how this “in-the-moment” openness translates into therapeutic practice. Is dialogue ultimately more of a one-way process in which the practitioner learns and explores the experiences, meanings and understandings of a person (and those close to them), but remains a bit of a “closed book” with the participants in the room having less opportunity to encounter the personhood of the practitioner? Or is it founded on the mutuality of peer relationships in which participants are continually learning of and from each other, and where both may be moved and changed through their encounter with the other? Although it is acknowledged that it may get “tricky when one person is paid” (Mead, 2014 p. 13), it is nevertheless core to IPS that Peer Practitioners put their whole selves “into the equation” (Mead, 2010a, p. 13) – hence a focus on the importance of relevant and appropriate self-disclosure as a key element of peer practitioners’ practice. In turn, this may provide a challenge – and an opportunity – for Open Dialogue practitioners who are not peers to be more open and disclosing of themselves within the therapeutic process.

A second area where there is significant shared ground between Open Dialogue and IPS is a phenomenological concern with meanings and interpretations – how we make sense of our experience and the possibility that there are always new ways of making sense that may emerge through connecting dialogically with others. Seikkula and Olson suggest that psychosis (and potentially other manifestations of mental distress) can involve a “temporary radical and terrifying alienation from shared communication practices: a “no-man’s land” where unbearable experience has no words and, thus, the patient has no voice and no genuine agency” (Seikkula and Olson, 2003, p. 409). Dialogue therefore involves reaching out to connect with others’ frames of expression and understanding “in order to develop a common verbal language for the experiences that remain embodied within the person’s … speech and private inner voices and hallucinatory signs” (Seikkula and Olson, 2003). This hermeneutical quest stands in radical opposition to more traditional mental health practices in which a dominant medical or psychological way of seeing can be imposed on what may seem dissident, anarchic or irrational. In order to safeguard the dialogical “between” space in which new language and understanding can emerge, a core principle of Open Dialogue is the tolerance of uncertainty and a willingness on the part of the practitioner to be comfortable in a place of “not knowing” for as long as it takes for meaning to emerge.

Taking a more explicit social constructionist stance, IPS invites people to “consider the possibility that there are many truths out there” (Mead, 2014, p. 6), inviting them to deconstruct dominant (and perhaps now habitual and internalized) ways of seeing and being. For example, instead of finding “ourselves falling into psychiatric assumptions about ourselves or others” (Mead, 2014), the uncertain and sometimes risky process of dialogue may create a space in which new and alternative meanings emerge, ones which may return to people opportunities for reclaiming voice and agency. Fundamentally, IPS is about conversation. It’s about how we … create new “knowing” through dialogue (Mead, n.d.). This co-creation of “new knowing” is given more of a political stance and purpose than in Open Dialogue: it is not just about breaking through the terrifying hermeneutic isolation of mental distress, it is also seen as purposive in bringing about social change – change that is predicated on hearing and learning from the suppressed meanings and experiences of those that may have undergone trauma, abuse and oppression (Mead, 2010b).

In finding ways to conceptualize ideas of plurality and indeterminacy, Open Dialogue draws upon the philosophical work of Mikhail Bakhtin. Bakhtin saw dialogical relations constructing everyday life, where meaning only emerges through dialogue. Identities and performances are always seen to be in flux and inherently unfinalized, continually open to being shaped by new encounters and experiences. From his analysis of Dostoevsky’s work, Bakhtin developed the concept of “polyphony” to describe a multi-voiced reality in which the “internally unfinalized consciousnesses” of participants play off each other, co-creating “a genuine polyphony of fully valid voices” (Bakhtin, 1984, p. 6, 176). What is spoken is a response to a previous utterance and, in turn, invites a new utterance to provide an answer. This sequence is never completed as new meanings arise whenever conversations recommence. A similar emphasis on the always-unfinished nature of human experience, and on the co-creativity in “playing off” one another, is to be found in IPS:

“Much like improvisation in music, IPS is a process of experimentation and co-creation, and assumes we play off each other to create ever more interesting and complex ways of understanding” (Mead, 2010b, p. 1).

In Open Dialogue, this idea of polyphony also draws upon social constructionist ideas of the validity of a plurality of viewpoints and subjectivities, rather than a search for a singular meaning or identity that can shut down or constrain the possibilities open to people. The role of the practitioner is to “guarantee that each voice becomes heard” (Seikkula, 2008, p. 489). The polyphony may comprise both the separate voices of interacting participants in an encounter and also their multiple internal voices or potential subjectivities, voices that may have become suppressed or fractured from one another through experiences of trauma and mental distress, or may simply reflect the more everyday ways in which people bring forward and articulate their different “selves” in relation to the various social contexts that they inhabit (Davies and Harré, 1990; Gergen, 1991).

Less explicit within Open Dialogue are understandings of power and inclusion. Connecting with the later work of Tom Andersen, Open Dialogue signals a shift from the hierarchical “professional – client” relationship characteristic of much clinical practice (and of earlier versions of systemic family therapy) to a heterarchy (Andersen, 1995, pp. 17–18) in which people and viewpoints are (in theory) seen as equally valid:

‘Within a “polyphonic conversation,” there is space for each voice, thus reducing the gap between the so-called “sick” and “well.” The collaborative exchange among all the different voices weaves new, more shared understandings to which everyone contributes an important thread. This results in a common experience which Bakhtin describes as “without rank”’ (Olson et al., 2014, p. 5).

However, concepts of heterarchy and “without rank” do not necessarily take account of how perceptions (and realities) of differential statuses within the room are likely to mean than power relations will be enacted, and some voices may potentially be privileged over others. From the perspective of professionals, it may be a challenge to give up positions of “knowing” and “power over” (see Chmnielowska et al., 2022; von Peter et al., 2023).

IPS offers a more overt consideration of power relations as experienced by those experiencing mental health difficulties:

“In communities of people who have been marginalized, there is an embedded sense of powerlessness that goes unrecognized. Identifying and talking about power dynamics is a beginning step toward breaking them down” (Mead et al., 2001, p. 139).

IPS recognizes “the power of language and labeling practices” in suppressing voices that challenge the dominant status quo of social organization, and how, even within therapeutic situations, there can easily be a re-emergence of oppressive patterns in which “various forms of power are used to blame, control decision-making, and recreate expert/patient type relationships” (Mead and MacNeil, 2014, pp. 3–4). This may be seen to connect with ideas of how the experience of recognition can be fundamental to social justice and emancipation (Fraser, 2000; Honneth, 2004). Being recognized by another person for “who we actually are” can feel profoundly validating and empowering, especially if our own sense of identity may appear a little uncertain or under threat, or in a state of emergence or transition. Conversely, being misrecognized (for example, being identified on the basis of one’s diagnosis) may be profoundly disempowering. Peer practitioners may be uniquely positioned to offer such recognition within a process of dialogic interaction and to understand how psychiatric diagnosis and other forms of social labeling can lead to such misrecognition.

In seeking to mobilize shared power through building mutually empowering relationships, IPS connects with feminist understandings of power as developed by Jean Baker Miller and colleagues, and particularly the work of Surrey, who characterized relational strategies of empowerment as involving “a mobilization of the energies, strengths, resources, or powers of each person through a mutual, relational process” (Surrey, 1991, p. 164) – something very different from more individualistic (and masculine-inspired) notions of self-determination or self-actualization. Her description of the operation of such power echoes the emphasis of both IPS and Open Dialogue on the generative nature of the “between” space in relationships and dialogue:

“The movement of relationship creates an energy, momentum or power that is experienced as beyond the individual, yet available to the individual… Neither person is in control: instead, each is enlarged and feels empowered” (Surrey, 1991, p. 168).

A final area of intersection between the two approaches is a focus on the person in their wider family, social and community contexts. Mead talks about the importance of moving away from unidirectional and dependent “service relationships” (which can characterize many mental health systems) to the reciprocity, the opportunity to give as well as to take, of being a “regular community member”:

“For many people relationships have become all about getting: telling your problem story and then getting help with it. There is little, if any, emphasis placed on giving back.. Service relationships are like a one-way street and both people’s roles are clearly defined. But in “regular” relationships in your community, people give and take all the time. No one is permanently on the taking side or the giving side. This exchange contributes to people feeling ok about being vulnerable (needing help) as well as confident about what they are offering” (2014, p. 5).

Whereas IPS places the key emphasis on reclaiming reciprocity and mutuality in personal relationships, including the relationship with the peer practitioner where vulnerability can be shared, Open Dialogue focuses more specifically on joining with the person’s family and social network itself. Taking a social network perspective is a core and defining principle of Open Dialogue – making sure that someone is not artificially separated from their relational environment but is always seen as a person-in-relationship-with-others. Families, and other key members of a person’s social network are always to be invited to the first meetings to mobilize support, not just around the person, but also for other members of the network who may be struggling to understand or deal with what is going on. Beyond this, connection may also be made with other agencies, such as housing or employment, who may be able to play a crucial role in maintaining (or creating) a place for the person in the wider social world.

The areas of commonality and difference in the conceptual underpinnings of Open Dialogue and IPS are summarized in Table 1. From this, it may be seen that, while there are powerful intersections between the conceptual framing of Open Dialogue and IPS, the latter cannot be subsumed into the former. Instead, it has the potential to bring an added conceptual dimension to underpin the practice of peer supported Open Dialogue.

**Table 1:** Open Dialogue and Intentional peer support – commonalities and differences.

	Open Dialogue	Intentional peer support
Authenticity of “I-thou” encounter and the emergence of new experiences and understandings of self and other in the “between” of the encounter	Central idea in conceptualizing the dialogical space in both OD and IPS	
Rejection of pre-planned interventions and solutions	Commonality and congruence between OD and IPS approaches.	
	Tolerance of uncertainty articulated explicitly as a practice principle	Emphasis on shift from *helping* the other to *learning with* the other
Phenomenological concern with questions of language, meaning, and interpretation	Shared emphasis in OD and IPS approaches	
	Emphasis on developing a common verbal language that is inclusive of the ways in which person may be expressing their distress	Need to challenge dominant and oppressive ways by which people may have come to see themselves and their mental distress – including labels and meanings constructed within the system of psychiatric care
Embracing plurality and indeterminacy	Congruence between OD and IPS approaches	
	Polyphony of fully valid voices	Emphasis on opening up the space for multiple truths
Approach to heterarchy and power in therapeutic discourse	Significant differences in how issues of power are addressed	
	Inclusive process in which all participants have a right to speak and be heard “without rank” – heterarchy instead of heterarchy	Greater recognition of embedded powerlessness of people within the discursive context of mental health services. Returning agency to people as a socio-political process based on solidarity, mutual learning and self-disclosure
Social networks and social relationships	Different but complementary emphases	
	Prioritization of including and working with the network rather than the individual in isolation	Emphasis on (re)learning how to do ordinary community relationships based on exchange and reciprocity

## From underpinning concepts to practice principles

Both Open Dialogue and IPS propose principles that seek to define and guide practice, drawing on their respective conceptualizations of the field that have been discussed above. The core ideas that define Open Dialogue practice are articulated within three of the seven Treatment Principles (the other principles, such as the provision of immediate help and psychological continuity, relate to more practical expectations around the organization of systems of care). These Principles are characterized as: a social network perspective; tolerance of uncertainty; and dialogism (Seikkula et al., 2003). IPS proposes principles that shift the focus on to learning (rather than helping); the relationship (rather than the individual); and hope and possibility (rather than fear) (Mead, 2010b, p. 1). Simply amalgamating these does not provide a coherent conceptual basis for Peer supported Open Dialogue – hence the rationale for our discussion with an Action Learning Set of peer practitioners to establish conceptual underpinnings that were grounded in their practice experience and would more clearly define the added value that IPS and the peer role can bring to dialogical practice.

A group of seven peer practitioners from across the ODDESSI research sites came together regularly to participate in an Action Learning Set. This provided an ongoing forum in which peer practitioners and researchers could bring issues and questions for reflective discussion – and for peers to share examples of their practice as a basis for reflective learning. With their agreement, a number of the discussions were recorded. These discussions covered a range of issues and experiences to do with developing and understanding the peer practitioner role in Open Dialogue teams. All of the peer practitioners were trained in Open Dialogue practice and shared some familiarity with IPS. They also brought a variety of experience in relation to peer support and activist roles.

Building on some of the earlier discussions in which peer practitioners had reflected on their use of self within network meetings, we introduced a discussion of “what is different in your way of connecting and being with people experiencing mental distress and family members from how you see other practitioners being with them?” Two sessions of the Action Learning Set focused specifically on what was different and additional that peer involvement could bring to network meetings – and how this might be captured in a set of practice principles. At the background of these discussions were ideas drawn from Open Dialogue and IPS, but, while these suggested some starting points, the main focus was on what emerged in the “between” space as participants shared and made sense of their practice experiences. Although discussions in earlier meetings of the Action Learning Set had tended to focus particularly on issues around self-disclosure, what emerged was a more nuanced sense that this was only one aspect of how peer practitioners might be able to offer something valuable on the basis of their lived experience. The discussion coalesced around certain key ideas which started to delineate the “additional” that peers could bring to network meetings. These suggested a conceptualization of the peer contribution based on the possibilities for *attunement; validation; mutuality and connection*; and *self-disclosure*. In turn, we have sought to translate these, and the reflections on experience of participants, into a set of preliminary principles to guide practice. From the discussions, there was considerable consistency in how understandings of attunement and validation came to be articulated. However, there was more diversity of viewpoints in relation to how mutuality and self-disclosure should be understood and practiced – and this has been reflected in the way that the principles have been formulated.

### Attunement

Responding to Seikkula’s challenge that “All that is needed is to be present and to guarantee that each voice becomes heard” (Seikkula, 2008, p. 489), peers may have an enhanced ability, based on surviving their own experiences of mental distress, to tune in to experiences that may be particularly hard to voice. As one peer put it, “Your antennae are more sensitive”. Attunement may involve picking up on and responding to a range of non-verbal and linguistic cues: “I do not know if it’s eye movement or body language or what it is, but it’s quite strange.. But then the language people use as well, I do pick up on that and I cannot work out how other people do not really notice it sometimes”. This process of attunement may be seen as generative, one that brings forward new understanding for both parties. This links to Mead’s analogy with musicians who can attune to and “play off” one another in an unfolding process of improvisation (Mead, 2010b, p. 1).

Within the wider polyphony of voices in the network meeting, it was felt that “if you have had similar experiences you’ll pick up on all sorts of things that others might not notice” – and the importance of self-awareness was recognized in mitigating against the imposition of the peer practitioner’s own “agenda” or experience. Prior experience of acute distress could make peers both more sensitive to pain and distress in the room, and also less likely to avoid it: “I think maybe I’m quite good at tuning into that pain, I can just relate to the pain and distress and maybe I’m less keen to cover it up… I’m curious about it”.

This enhanced ability to attune may go wider than simply picking up on what may seem personally familiar or resonant. Peer experience can give a heightened ability to sense and connect with feelings even when the actual content of experience may be very different: “I can really tune into the mum even though I have not had a child with psychosis I can just really feel it somehow.” Being attuned can mean responding to cues to bring network members into the dialogue. By tuning in to the mother’s previously hidden voice in this way, “the mother and son began to then talk for the first time about stuff that had not been addressed but was clearly important.” An enhanced ability to attune may not just apply in relation to connecting with a particular person; it may also apply to “reading a room” for signals of the not yet spoken – which can add “an extra dimension in terms of understanding what’s happening in the wider group, as well as what may be the voices in that individual.”

From this discussion, we propose the following articulation of a new Practice Principle for Peer Practitioners which provides an initial characterization of what may be possible through attunement:

Through their personal lived experience, peer practitioners can bring a particular attunement to the emotions of others in the room, as well as a developed sense of awareness of, and sensitivity to, the implicit and explicit language that they may be using.

### Validation

Seikkula and Olson (2003) highlight the isolation and hermeneutic exclusion of people experiencing their own unique manifestations of mental distress for which they have no language – and hence having only very limited possibilities for this experience to be recognized or understood by others. Buber uses the term “confirmation” to describe a process in which our own unique subjectivity (and humanity) can only be actualized when it is accurately mirrored, and returned to us, through our encounter with another person – an inherently reciprocal process in which the other person allows themselves to be open to receive our confirmation of their unique and present subjectivity. Connecting more with the social action agenda of IPS, such “confirmation” may also be viewed in terms of recognition as a step toward the attainment of social justice (Fraser, 2000; Honneth, 2004) – a struggle that may be taking place within a wider context of potential stigma, oppression and misrecognition, both by the mental health system and wider society. As one peer practitioner put it, “it’s just like communities of people who have shared experience who do not start from a place of disbelief”.

By virtue of their own lived experiences of mental distress, and perhaps also their own experiences of invalidation and misrecognition, peer practitioners are uniquely positioned in terms of being able to offer a mirroring that can affirm that the experiences of the person or network member are real, and that they deserve to be acknowledged. They can be sensitized to the potential inimical effects of certain psychiatric practices (such as diagnosis) on recognition and validation: “when you do not look at that … you meet the person that you are asked to work with, it’s strange.” Unusual experiences associated with psychosis can be understood and normalized, rather than pathologized: “sometimes it’s the way we connect that makes people feel what they are going through is real.” By their very presence, and the potential grounding of reflections in their own experience, peer practitioners may be more able to assure the person that their voice is legitimate and credible. If people feel safe and supported to speak, they may share experiences they have never expressed before. In particular, peer practitioners can be in the position to hear and acknowledge people’s extremes of anguish or despair: “recognizing somebody’s hopelessness [can be] very validating – you are actually validating who they are rather than who the [mental health services] would like them to be”. Some people take longer than others to navigate their personal journey, while others do not change. Peer practitioners can provide legitimation that where people are can be “a valid place to be, like it’s okay”. Paradoxically, by taking away the pressure to get better in response to the expectations of others, such validation may give people power and ownership in relation to their experience – a sense of empowerment which, in turn, can form a foundation for recovery (Leamy et al., 2011).

For someone who may be struggling with a multiplicity of internal voices or conflicting emotions, it may be important not just that they feel validated as a person, but also that “the polyphony of the voices all talking together” is also recognized as valid and important. This can be the start of a process of “enabling people to start feeling that they can talk about these things, which I think has been really important about normalizing it and maybe taking away some of the stigma.” Again, it can be the lived experience of the peer practitioner that offers a particular ability to be “at ease” with the different elements of a fractured subjectivity – and a recognition that it is only through being able to put these elements out into the open in a safe and validating space that the complexity of their distress can be fully heard and acknowledged. By offering such recognition, peer practitioners establish connections that in some way alleviate pain and isolation or engender hope: “there’s a kind of magic where you do actually feel once you say it and express it and the other person hears it, it does actually slightly leave you, do you know what I mean? It’s quite strange.” In turn, this can then provide the opportunity for a process of healing and reintegration – no longer having to hide these elements in an internal world of terrifying isolation, but instead receiving validation, potentially not just from a peer practitioner, but also from those in their network that matter to them. It is through facilitating this wider process that Peer-supported Open Dialogue can create “a space for them to be in the world as a valid person” – something that may be seen as a cornerstone for recovery (Bradshaw et al., 2007).

Another aspect of validation that can be important can be where people have felt that their experiences have been invalidated, not just by people in their family or social networks, or by wider social attitudes, but by mental health services themselves: “it becomes very difficult to talk about the harm that has been experienced by those systems if it’s not going to be at all validated by anyone around me.” This difficulty in speaking out and being heard about the harm caused by systems may be an ongoing issue, not just for people receiving services but also for peer practitioners working in such services.

Building on these emerging insights, we propose the following conceptualization of validation as a second Practice Principle for Peer-supported Open Dialogue:

By explicitly and implicitly using their lived experience, peer practitioners can validate and provide recognition for the current experience of people who may be facing misrecognition by others, or coming to doubt the validity of their thoughts and feelings. In turn this can offer empowerment and engender hope by enabling people to reclaim their sense of self-worth and self-belief.

### Connection and mutuality

Peer practitioners are perhaps uniquely positioned to understand the nature (and challenge) of connecting when one party is experiencing mental distress. As one peer practitioner put it, connecting can be more “spontaneous” and “instinctive”: “…there can be no hard or fast rules and if you think about rules…It’s not going to be authentic, it’s not going to be spontaneous.” This may more easily enable a “here and now interaction” (Galbusera and Kyleso, 2017, p. 3), in contrast to clinical practitioners whose openness to make such an intensely personal connection may be more constrained by “baggage” in terms of role expectations and previous training in relation to professional boundaries. The risk of connecting may also be perceived differently by the person experiencing mental distress, if the person who is seeking to connect with them is already perceived as someone who might know and understand some of their vulnerability. It is therefore possible that the immediate “getting to know” can be framed within a mutuality of risk taking – hence making it easier to build trust.

Their experience may afford peer practitioners a greater awareness that connection requires time and space – in contrast to more traditional clinical practice which can be characterized by controlling interactions and faster treatment trajectories: “I think with some [clinical] practitioners just to sort of like get it done, you know, sort of move on.” Such a professionally driven urge to act contrasts with the key Open Dialogue practice principle of tolerance of uncertainty (Seikkula et al., 2003), and peer practitioners may find it easier simply to “be with” and build a deeper trust rather than (however unconsciously) push for solutions. Because of their own experience of “being with” their own distress, peer practitioners may be better able to “be with” a person in acute distress and less afraid to connect with them in that space: “you could be more…comfortable being with someone who’s quite acutely stressed because you have been there, and you have survived it… Whereas others that maybe had not experienced that intensity of the stress themselves were just more scared of it.” In this way, the presence of a peer co-worker in a network meeting may, in itself, offer permission to clinicians to leave behind this aspect of their background and hence be better able to stay with distress rather than seek to cure it.

Although there are no formal rules for connection, it can be important for peer practitioners to be at the first Network Meeting where the initial connection can be made with the person at time of crisis: “I’ve felt the most connection with people where I’ve been invited right at the beginning of the crisis, and I’m kept within that network.” Galbusera and Kyleso (2017) emphasise the importance of the core organizational principle of psychological continuity, “which means that the responsibility for the client’s health care rests with the same reference professionals for the duration of the whole treatment” (p. 2).

For the peer practitioner, shared experiences of social oppression can result in connection through a sense of solidarity: “it might not be called that in the room … but for me, that’s a sense of like political consciousness, like a connection of solidarity of oppression.” Humor can be a way of connecting around (and resisting the negative impacts) of such experiences – including those linked to their receipt of services: “Sometimes I feel connected in a slightly mocking position of services in connection with the person, in a sense of like a slightly shared smile.” While peer practitioners are often aware of the tensions with their own position as paid workers, they are not bound by the same statutory responsibilities as their clinical practitioner counterparts. This may allow for greater openness to connection and developing relationships based on a greater similarity in their experiences of the operation of power: “I do not think any peer workers have any statutory responsibility around incarceration or sectioning or anything like that I think affects the ability to be with…that allows me a certain level of proximity to somebody that they cannot do.”

When connection happens the energy between the people in the room can shift: “there’s almost a tangible change in the energy environment in the room when there’s a real connection between the person with lived experience and the person in distress at that time.” Connection can happen through empathy and shared feeling which can also affect other Network members: “I think there’s a huge amount about feeling the pain but also having the empathy. And having been there and felt it I think it’s a strong connection to some of the network members.” The feeling of connecting itself can be difficult for peer practitioners to describe but words like “magic”, “chemistry” and “uncanny” seemed to fit for them. Seikkula observes that in dialogue, “living persons emerge in real contact with each other … without controlling and deliberating on their behavior in words” (Seikkula, 2011, p. 186). Peer practitioners can give insight into this deeper experience of connecting: “So, it’s the non-verbal utterances and sometimes we do not even have to say what we are feeling or what we are experiencing or what we are connecting with”.

Although strongly emphasized in IPS, ideas around mutuality and equality of exchange do not always fit easily with the peer practitioner role in Open Dialogue – and this emerged as an area of potential tension and dissonance for the peer practitioners. As one put it, “the mutuality thing, I think gets used in the way that we talk about Open Dialogue quite a lot. I feel like – I do not know, I feel conflicted about it”. Another voiced their concern in more political terms:

“For me, ‘mutuality’ means social change because then you actually are helping each other and there’s some kind of change happening and there’s a sense of solidarity and building and changing. For me, that’s why it can never be a social movement because it’s not actually mutuality, we are a health provider.”

Others articulated a sense in which both peer practitioner, and the person with whom they were connecting, could both be moved in a real way by the other: “It’s also connecting with the experience of having the experience”. This fits with Hartmut Rosa’s conception of “relations of relatedness” which are characterized by a resonance in which, “in the course of a given interaction, [people] are touched or affected by an Other or by others and, moreover are themselves capable of touching or affecting others” (Rosa, 2019, p. 179). As another peer practitioner put it, they would be open to personal learning “from what I can see or the person tells me”. This sense of being moved by (and learning with) the person connects both with a key principle of IPS and with Galbusera and Kyleso’s articulation of a “responsive response” in which the practitioner does not disappear as a subject in the dialogic encounter: “Listening and acknowledging the other person are not merely about recognizing the other in the sense of passive witnessing but about what we might call with-ness, the readiness of stepping together into the interaction” (Galbusera and Kyleso, 2017, pp. 5–6). It involves feeling able to bring one’s whole personhood (and not just some construction of a professional self) into the interaction. Although practitioners from professional backgrounds also report how they have been moved in dialogic encounters (Taylor et al., 2023), such an ability to “step together” into a space of shared learning may come a little more easily when entering the interaction from the orientation of peer rather than professional. However, the presence of a peer as co-worker may also enable clinical practitioners to take the risk of bringing more of their whole personhood into the interactional space.

We propose, as the third Practice principle, the following characterization of the approach to connection and mutuality that peer practitioners can bring:

Drawing upon their personal experience, peer practitioners can connect with a person and members of their network by being open to a more mutual relationship in which they can share how they themselves are moved by the emotions and experiences that are expressed. In doing so, they can show how there is no need to be afraid of intense emotions, and thereby keep the focus on connecting and being with people in their experience, rather than reaching for solutions. This may help to build a supportive “between” space in which people can find their own ways of moving forward.

### Self-disclosure

Self-disclosure is not a new concept to Open Dialogue, and some practitioners may choose to share life experiences during a network meeting. This is mirrored in Peer supported Open dialogue, which encourages practitioners from all disciplines, to share life experiences, when they feel it is safe, helpful and appropriate to do so within a therapeutic meeting. Such an approach is reflected in Jourard’s broader “self-disclosure” theory where the therapist “checks this [self-disclosure] by common sense and judgment, and he limits it to an openness of himself *in that moment*” (Friedman, 1985, p. 10). In practice, while there are studies on the benefits and challenges of self-disclosure from professionals (Knox et al., 1997; Hill et al., 2018), for many staff within statutory mental health settings, self-disclosure is a new concept and approach. With the number of people employed to use their personal experience as an explicit part of their role is growing, self-disclosure is becoming increasingly visible in health care settings (Ahluwalia, 2018; Byrne et al., 2022).

Within the context of Open Dialogue, peer practitioners felt self-disclosure could have a powerful impact in network meetings. As one practitioner reflected “I think the more concerned we get with ‘should I or should not I disclose’ and all of that, it stops us from being fully human, fully authentic, and effective.” Another peer reflected “It seems like almost that just happened in that conversation, someone’s experience really relating to someone else’s experience.” Peer practitioners described using their intuition and discernment before choosing to self-disclose directly in a network meeting or as part of a reflecting conversation with a colleague. One peer practitioner commented “There might be things that I might to say, but in Open Dialogue, it’s also about discernment. What would be helpful to share now? And if something has actually triggered me to do with my own lived experience, I guess I go back to a reflection.”

The presence of peer workers in mental health teams can create a culture in which staff may feel safe and empowered to share their lived experience and actively use this within their own practice (Byrne et al., 2022). When self-disclosure is used responsively and appropriately, it can encourage others present in the meeting to share personal experiences (Truong et al., 2019). One peer practitioner reflected ‘I felt just recently that when I disclose, sometimes it’s in a reflection and the other practitioner will immediately say, “yes, I’ve got that experience as well”… I think they really do want to talk about their own experience and its sort of like opened it up’.

However, any moves toward self-disclosure may be taking place within a pre-existing culture in which upholding personal boundaries was seen as a cornerstone of professional practice. This may explain why, in some cases, peer practitioners noticed mixed responses from colleagues when they self-disclosed in a network meeting. One peer practitioner shared “We have a valid, kind of almost overt part to use our lived experience. I think a lot of the confusion and fear is because you divulge stuff instinctively in the meeting. Very often I’ve seen colleagues looking uncomfortable.” Within a working context in which self-disclosure did not always feel supported, another peer practitioner had chosen to become more reticent about offering this: “When I first started, I did try and bring in more self-disclosure. I do not so much now, I think because it does get latched on to and I think I’ve noticed it does change things quite a lot”.

While the value of self-disclosure was widely acknowledged by the peer practitioners, the emotional cost to the person disclosing was also apparent: “It’s the level of self-disclosure and the emotional energy it takes and what it takes out of you”. Another reflected, “Most of the time it has a really good outcome. But you absorb all of that and it drains you completely”. However, one peer identified disclosing in a supportive space can reduce emotional toil “I did not feel exhausted sharing in that space, it was a really supportive space.”

What is apparent, is that the process of self-disclosure is complex and nuanced. Self-disclosure brings a level of vulnerability to the person disclosing and they need to consider their emotional safety as well as the safety of others in the room. The orientation of co-workers can make a difference whether the peer practitioner may feel safe enough to disclose or not. Self-disclosure is proposed as a fourth Practice Principle for peer practitioners in Open Dialogue:

The title of peer practitioner already constitutes a level of self-disclosure, indicating that a person has their own experience of mental health difficulties alongside wider life experience. In network meetings, peer practitioners should use their discernment and intuition to assess whether self-disclosure would or would not be helpful in supporting or bringing out other voices in the room – and should only do this when they feel it is safe and helpful in doing so.

## Conclusion

This paper offers a conceptual framework organized around a set of practice principles to underpin Peer supported Open Dialogue (see Table 2). These principles have been developed out of discussions with peer practitioners working in Open Dialogue teams located in ODDESSI trial sites in England and are grounded in their practice experience. In their paper exploring peer support and shared decision making in Open Dialogue, Chmnielowska et al. (2022) argue that “clarifying the core values and principles of the PSW [peer support worker] in OD [Open Dialogue] will ensure that, as peer support grows, it grows with integrity…” (p. 4). Here we offer a basis for the further exploration of a set of core principles. The four practice principles presented here - attunement, validation, connection and mutuality, and self-disclosure – may be seen to build on core ideas inherent in IPS and Open Dialogue.

**Table 2:** Four practice principles that develop a conceptualization of the additional contribution that peer practitioners can bring to Open Dialogue.

Attunement	Through their personal lived experience, peer practitioners can bring a particular attunement to the emotions of others in the room, as well as a developed sense of awareness of, and sensitivity to, the implicit and explicit language that they may be using.
Validation	By explicitly and implicitly using their lived experience, peer practitioners can validate and provide recognition for the current experience of people who may be facing misrecognition by others, or coming to doubt the validity of their thoughts and feelings. In turn this can offer empowerment by enabling people to reclaim their sense of self-worth and self-belief.
Connection and Mutuality	Drawing upon their personal experience, peer practitioners can connect with a person and members of their network by being open to a more mutual relationship in which they can share how they themselves are moved by the emotions and experiences that are expressed. In doing so, they can show how there is no need to be afraid of intense emotions, and thereby keep the focus on connecting and being with people in their experience, rather than reaching for solutions. This may help to build a supportive ‘between’ space in which people can find their own ways of moving forward.
Self-disclosure	The title of peer practitioner already constitutes a level of self-disclosure, indicating that a person has their own experience of mental health difficulties alongside wider life experience. In network meetings, peer practitioners should use their discernment and intuition to assess whether self-disclosure would or would not be helpful in supporting or bringing out other voices in the room – and should only do this when they feel it is safe and helpful in doing so.

A clearer conceptualization of the nature of the peer contribution may be seen as crucial in development of Open Dialogue services where currently the specific challenges and opportunities associated with the peer role may not be well understood – both by peers themselves and by professional colleagues and services more widely. For peers, the proposed principles provide a clearer articulation of what they may be able to bring to a network meeting on the basis of their lived experience. These may be particularly useful in training and supervision, so as to maintain and enhance the integrity of the role. They may also be important in providing role clarity within clinical teams and improving collaboration with colleagues. A clearer articulation of the peer contribution also has implications for recruitment and role specification – and there would seem to be a strong argument that the enhanced opportunities for dialogical connection that peers can bring should be made much more widely and consistently available across services.

Having lived experience of emotional or mental distress can mean greater tolerance of uncertainty, a readiness to navigate the complexity of distress or unusual experiences and perhaps more confidence in “being with” and connecting in a way that offers more of an experience of mutuality, rather than an (unspoken) sense that it remains the duty of professional practitioners to implement solutions for and on behalf of people. However, this conceptualization illuminates how the potentially greater use of self may have implications in terms of sustaining longer term wellbeing. This requires consideration of what Scott (2011) calls “love labor”, which can both be intensely rewarding, but can also be emotionally challenging – and hence the importance of tailoring opportunities for supervision and intervision that provide peer practitioners with a safe and protected reflectional space. Perhaps most of all, it provides a basis for recognition by professional colleagues of what, more specifically, peer practitioners may be able to bring to dialogic encounters – and the possibilities that this may open up. Currently there can be contradictory expectations within services that, on the one hand, public self-disclosure may be seen as an expectation of the role while, simultaneously, professional colleagues may show discomfort with the practice as it may challenge their understandings of professional boundaries. These principles provide a broader basis for understanding the range of “added value” that lived experience can bring, in terms of an enhanced ability to offer attunement, validation and mutuality in connection, while emphasizing that they may need to use both intuition and discernment in order to judge when and how self-disclosure may free up or facilitate the dialogue in the room. These principles may also be of value to practitioners from professional backgrounds in providing a framework within which they could also feel more confident in giving and sharing of themselves, and drawing on their own lived experience of challenge or distress. There is a need to work with all Open Dialogue practitioners to further understand how self-disclosure and vulnerability are experienced, so guidelines for navigation can be co-produced.

The translation of these principles into mainstream therapeutic practice may be challenging as they can run counter to many conventional mental health practices and a dominant biomedical culture. Peer practitioners work within a system that may have caused them harm and their experiences need to be recognized and validated. As the value and contribution of the peer practitioner in Open Dialogue becomes better understood and appreciated, the possibility of gradual, transformational change opens up. Peer practitioners can help provide impetus for a cultural shift within their team that, in turn, can impact on the wider service. Acting as co-facilitators with clinical colleagues, they can create a space where different ways of “being with”, validating and normalizing can be witnessed by colleagues and model ways to hear, acknowledge and respond to the voice of distress.

## Author contributions

All authors listed have made a substantial, direct, and intellectual contribution to the work and approved it for publication.

## Funding

The research was funded by the National Institute for Health Research Grant number RP-PG-0615-20021.

## Conflict of interest

The authors declare that the research was conducted in the absence of any commercial or financial relationships that could be construed as a potential conflict of interest.

## Publisher’s note

All claims expressed in this article are solely those of the authors and do not necessarily represent those of their affiliated organizations, or those of the publisher, the editors and the reviewers. Any product that may be evaluated in this article, or claim that may be made by its manufacturer, is not guaranteed or endorsed by the publisher.
